# *Duchesnea indica* Extract Attenuates Coal Fly Ash-Induced Inflammation in Murine Alveolar Macrophages through the NF-Kappa B Pathway

**DOI:** 10.1155/2021/5546052

**Published:** 2021-06-04

**Authors:** H. M. Arif Ullah, Yuan Yee Lee, Sung Dae Kim, Man Hee Rhee

**Affiliations:** Department of Veterinary Medicine, College of Veterinary Medicine, Kyungpook National University, Daegu 41566, Republic of Korea

## Abstract

*Duchesnea indica* is known as false strawberry, is found in East Asia, and has numerous biological properties. The aim of this study was to investigate the anti-inflammatory effect of *Duchesnea indica* extract (DIE) on coal fly ash- (CFA-) induced inflammation in murine alveolar macrophages (MH-S). Following the induction of inflammation in MH-S cells by CFA, nitric oxide (NO) was measured to evaluate the anti-inflammatory property of DIE. Cell viability and inflammatory gene expression were analyzed using polymerase chain reaction (PCR). The inflammatory pathway in MH-S cells was determined via western blotting and immunofluorescence (IF) analysis. Finally, the major components of the DIE were identified and separated through ultra-performance liquid chromatography (UPLC) and gas chromatography-mass spectrometry (GC-MS) analysis. Our results showed that the DIE dose-dependently inhibited the CFA-induced NO production in MH-S cells. Moreover, the DIE could suppress the CFA-induced proinflammatory mediators, such as cyclooxygenase-2 (COX-2) and inducible nitric oxide synthase (iNOS). In addition, the inhibitory effect of the DIE on proinflammatory cytokines, including interleukin-6 (IL-6), IL-1*β*, and tumor necrosis factor-*α* (TNF-*α*), was detected with PCR. Moreover, the effect of the DIE on the nuclear factor-*κ*B (NF-*κ*B) pathway in CFA-activated MH-S cells was measured via western blotting. Furthermore, the inhibition of the phosphorylated NF-*κ*B (p-NF-*κ*B) translocation was analyzed using IF assay. The findings of this study indicated that the DIE potentially inhibited the CFA-induced inflammation by blocking the NF-*κ*B inflammatory signaling pathway in MH-S cells and that the DIE might contain favorable anti-inflammatory compounds which may be effective in attenuating lung inflammation.

## 1. Introduction

Coal fly ash (CFA) is a major environmental factor of air pollution, which is produced regularly due to industrial activities and the use of motor vehicles in the urban areas [1]. CFA consists of liquid and solid particulate matters that vary in size and origin, including coal, combustion particles, and asbestos [1, 2]. Nanoparticles can retain for a long period of time in the atmosphere and easily move in the air. Chronic exposures of CFA can cause accumulation of sediments in the lungs and lead to serious adverse effects, such as inflammation in the alveolar cells [3, 4]. Moreover, nanoparticles are responsible for many respiratory diseases, such as asthma, bronchitis, chronic obstructive pulmonary diseases (COPD), and lung carcinoma [5].

Inflammation is an important biological response to harmful stimuli and the first line of defense in the immune system [6]. Alveolar macrophages (AM) are considered one of the defensive cell populations in the lungs and facilitate regulating lung inflammation and host defense protection against airborne nanoparticles [7]. After CFA invades the airway of the lungs, AM are recruited, resulting in the production of inflammatory mediators and cytokines, which is the initial response of the immune system [8]. CFA-induced excessive proinflammatory cytokines are considered one of the major causes of lung diseases. They can be regulated by the activation of the transcription factors, such as nuclear factor kappa-B (NF-*κ*B) signaling cascade [9, 10].

*Duchesnea indica* is a perennial plant that belongs to the Rosaceae family and is widely distributed in Asian countries especially in Bangladesh, China, Japan, and Korea [11]. It has been extensively used as a folk medicine in Asia for its unique biological properties including antioxidative, anti-inflammatory, antimutagenic, and anticancerous properties [12, 13]. Several studies have demonstrated that some phenolic compounds, such as phenolic acid, flavonoids, and ellagic acid, are the main pharmacologically bioactive constituents in *Duchesnea indica* [11]. It has been shown that the *Duchesnea indica* extract (DIE) downregulates cervical cancer through apoptosis and cell cycle arrest [14]. Moreover, previous studies have reported that the DIE protected the skin fibroblast of humans against hydrogen peroxide-induced cytotoxicity and inhibited the growth of human cancer cells in vitro [15]. Thus, traditional medicines have been considered by the patients due to their therapeutic effects and bioactive ingredients [16].

In this study, we aimed to investigate the anti-inflammatory effects of the DIE on CFA-induced inflammation in MH-S cells. Taken together, our results demonstrated that the DIE can prevent the inflammation in CFA-activated MH-S cells, and such activity is mediated by inhibiting the expression of proinflammatory mediators and cytokines.

## 2. Materials and Methods

### 2.1. Chemicals and Reagents

The following chemicals and reagents were utilized: Roswell Park Memorial Institute Medium (RPMI), fetal bovine serum (FBS), penicillin-streptomycin, and Dulbecco's phosphate buffered saline (DPBS) (Welgene, South Korea); CFA and 3-(4,5-dimethylthiazol-2-yl)-2,5-diphenyltetrazolium bromide (MTT) (Sigma-Aldrich, St. Louis, MO, USA); Oligo-dT (Bioneer oligo synthesis); COX-2, iNOS, TNF-*α*, IL-1*β*, and IL-6 primers (Bioneer, Daejeon, South Korea); dimethyl sulfoxide (DMSO) (Sigma-Aldrich, St. Louis, MO, USA); TRIzol reagent (Invitrogen, Carlsbad, CA, USA); Pro-prep (iNtRON biotechnology, South Korea); bovine serum albumin (BSA) (Thermo Fisher Scientific, South Korea); specific antibodies used for western blot, including COX-2, iNOS, p-IKB, p-NF-*κ*B, *β*-actin, and HRP-conjugated secondary antibody (Cell Signaling Technology, Danvers, MA, USA); secondary antibody used for immunofluorescence (anti-rabbit IgG Fab2, Alexa Fluor 555, Molecular Probes). All other chemicals and reagents were the highest quality available.

### 2.2. Preparation of the *Duchesnea indica* Ethanol Extract (DIE)

We purchased dried *Duchesnea indica* leaves and stems from the company, ground them into fine coarse powder, and extracted them in 70% ethanol (1-part dry weight of plant to 20-parts of solvent ratio) using a heating mantle (Model: MS-DM604, MTOPS, South Korea) at 80°C for 2 h. Then, the extract was filtered using the Whatman filter paper, followed by extract condensation using the rotary evaporator system (Heating bath-B100 and Rotavapor-R100, BUCHI, Switzerland). Finally, the crude extract was frozen overnight at −70°C and lyophilized using a freeze dryer (Model: FDU-7012, Operon, South Korea) to obtain dry fine powder. The yields were weighed and preserved at −30°C for use in experiments. At the time of the experiment, the powder was dissolved in DMSO with different doses of DIE (12.5, 25, 50, and 100 *μ*g/mL).

### 2.3. UPLC-QTOF-MS Analysis of DIE

UPLC analysis of the DIE was performed using ACQUITY UPLC^TM^ (Waters Corp., Milford, MA, USA), equipped with a binary solvent delivery system and coupled to the quadrupole time-of-flight mass spectrometer (Q-TOF Premier^TM^, Waters Corp., Milford, MA, USA) equipped with an auto-sampler and a UV detector. Briefly, the DIE (2 *μ*L) was injected into the ACQUITY UPLC BEH C18 chromatography column (2.1 × 100 mm × 1.7 *μ*m). The column temperature was fixed at 35°C, and the flow rate was 0.4 mL/min. The chromatographic gradient consisted of mobile phases: (A) water with 1% formic acid and (B) acetonitrile with 1% formic acid. The gradient duration was optimized: 0 min, 10% B; 0–11 min, 10%–90% B; 11–11.5 min, 90%–100% B; 11.5–13.5 min, 100% B; and 13.5–15 min, back to 10% B. The mass spectrometer condition was a negative ion mode with the capillary and cone voltages being 2.3 kV and 50 V, respectively. N_2_ was used as a desolvation gas. The source temperature was 100°C, and the desolvation temperature was 350°C. Leucine-enkephalin was used as a reference compound (m/z 554.2615) in the form of a spray.

### 2.4. GC-MS Analysis of DIE

GC-MS analysis of the DIE was performed using the Agilent 7890A GC (Agilent Technologies, Santa Clara, CA, USA). The GC-MS device was equipped with a 30 m × 0.25 mm (i.d. DB-5MS) chromatography column and the Agilent 5975C mass selective detector to separate and quantify the compounds of DIE. The extract was injected at 250°C. The temperatures for the transfer line and source were 280°C and 230°C, respectively. The column temperature was set at 70°C as an initial temperature for 1 min and raised to 300°C at a rate of 5°C/min, with duration at a final temperature of 300°C for 30 min. The mass spectrometry was acquired via electron ionization and scan modes. The helium gas was used as a carrier gas with a constant flow rate (1 mL/min).

### 2.5. Cell Culture

The MH-S cell line, originating from the American Type Culture Collection, was cultured in RPMI supplemented with 10% FBS, 100 IU/mL penicillin, and 100 *µ*g/mL streptomycin sulfate (Welgene, South Korea). The incubation conditions were maintained at 37°C and 5% CO_2_ during culture and treatment conditions.

### 2.6. Nitric Oxide (NO) Assay

NO was measured using the Griess reagent A (0.2% N-ethylenediamine dihydrochloride) and Griess reagent B (2% sulfanilamide in 5% phosphoric acid) reaction methods. Briefly, the MH-S macrophages were seeded in a 24-well plate and incubated with or without CFA (2.5 *μ*g/mL) in the absence or presence of the DIE (12.5, 25, 50, and 100 *μ*g/mL) at indicated concentrations for 18 h. The Griess reagents (100 *µ*L) were added with cell culture supernatants (100 *µ*L) and incubated for 5 min at normal condition. Then, absorbance was measured in a microplate reader at 540 nm (Versamax, Microplate Reader, Molecular devices, CA, USA).

### 2.7. Cell Viability Assay

To determine the cytotoxicity of the extract, a cell viability assay was measured as described using 100 *µ*L/well of 3-(4,5-dimethylthiazol-2-yl)-2, and 5-diphenyltetrazolium bromide (MTT) reagent was added to the culture medium [17]. After 2–3 h of incubation at 37°C in 5% CO_2_, the supernatants were discarded, and dimethyl sulfoxide (DMSO) (100 *µ*L/well) was added and then incubated at room temperature with shaking for 10 min. Finally, absorbance was analyzed using microplate reader at 560 nm (Versamax, Microplate Reader, Molecular devices, CA, USA).

### 2.8. RNA  Extraction, Reverse Transcription-Polymerase Chain Reaction (RT-PCR), and Real-Time qRT-PCR

PCR analysis was conducted in accordance with the previously reported method [18]. The murine macrophages (MH-S) were treated with or without the DIE (12.5, 25, 50, and 100 *μ*g/mL) for 30 min at indicated concentrations, followed by the CFA stimulation (2.5 *μ*g/mL) for 18 h. RNA was collected from cells using TRIzol reagent (Invitrogen, Carlsbad, CA, USA). Two micrograms of total RNA was annealed with Oligo-dT (Bioneer, South Korea) for 10 min at 70°C, cooled for 5 min in ice, and then reverse-transcribed using reverse transcriptase premix (Bioneer, South Korea) in 20 *µ*L of reaction mixture for 90 min at 42.5°C on a thermocycler. To inactivate the reverse transcriptase, the reaction was terminated at 95°C for 5 min. RT-PCR was performed using cDNA obtained from RT reaction in a PCR premix (Bioneer, South Korea). Moreover, the PCR products were electrophoresed on agarose gel (1%) stained with ethidium bromide. The band was visualized using ImageQuant LAS 500 (GE Health Care Life Sciences, South Korea). The intensity of the band density was normalized to GAPDH. Real-time PCR was performed using SYBR green.  Table 1 shows the RT-PCR and real-time PCR primer sequences.

### 2.9. Western Blot Analysis

Western blot analysis was conducted in accordance with the previously reported method [10]. MH-S cells were untreated (control group), treated with only CFA (2.5 *μ*g/mL), and treated with the DIE (12.5, 25, 50, and 100 *μ*g/mL) in the presence of CFA (2.5 *μ*g/mL). The proteins were extracted from the cells with the protein extraction solution, Pro-Prep (iNtRON biotechnology, South Korea). Then, the proteins were determined using the protein measurement solution, PRO-MEASURE assay kit (iNtRON biotechnology, South Korea). The cell lysates were then subjected to SDS-PAGE (10%) and transferred onto the PVDF membranes (Millipore, Immobilion-P, Billerica MA, USA). Membranes were blocked for 1 h in 5% (*w*/*v*) skim milk and 0.1% (*v*/*v*) Tween-20 in TBS. Then membranes were incubated with different primary antibodies overnight at 4°C. After washing, 1 h of incubation with horse radish peroxidase- (HRP-) labelled secondary antibody (1 : 3000 dilution, Cell Signaling) at room temperature was performed. Proteins were detected using enhanced chemiluminescence (ECL) solution (Supex, Daegu, Korea). Immunoblots were quantified using the ImageJ software.

### 2.10. Immunofluorescence Staining

Immunofluorescence (IF) assay was done as described [19]. MH-S cells were washed with DPBS and fixed with 4% paraformaldehyde (PFA) for 10 minutes. Moreover, the cells were permeabilized with 0.2% triton X-100 in TBS (TBST) for 10 minutes and washed with TBST for 5 minutes every three times. Using 2% BSA, the samples were blocked for 1 h, and primary antibody rabbit anti p-NF-*κ*B (Cell Signaling Technology, Danvers, MA, USA) was applied to the cells at 4°C overnight. The cells were washed with TBST for 5 minutes every three times. The samples were incubated with secondary antibody (anti-rabbit IgG Fab2, Alexa Fluor 555, Molecular Probes) for 1 hour in the dark, and after washing with TBST three times, the samples were mounted using ProLong Gold Antifade Reagent with DAPI to visualize the nuclei (Cell Signaling Technology, Danvers, MA, USA) and analyzed via confocal microscopy (ZEISS).

### 2.11. Statistical Analysis

Data were analyzed by one-way ANOVA or unpaired Student's *t*-test followed by Dunnett's multiple comparison tests using the GraphPad Prism software. Data are presented as mean ± SEM. The statistical significance is denoted as ^*∗*^*p* < 0.05, ^*∗∗*^*p* < 0.01, and ^*∗∗∗*^*p* < 0.001 or ns = not significant.

## 3. Results

### 3.1. Active Compositions of the DIE Based on the UPLC-QTOF-MS and GC-MS Analyses

**Figure 1:**
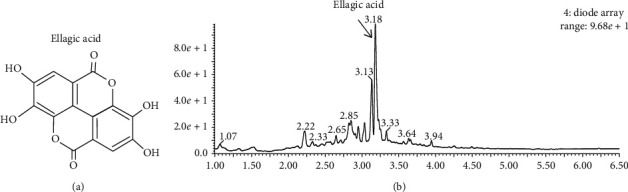
Chemical compositions of the *Duchesnea indica* extract (DIE). (a) Chemical structure of ellagic acid. (b) Ellagic acid was identified via UPLC-QTOF-MS analysis as a major compound, and the retention time was 3.18 min.

Active compounds in the DIE were determined via ultra-performance liquid chromatography (UPLC) analysis. Based on the results of the UPLC-QTOF-MS analysis, the main active compound of the DIE was ellagic acid (Figure 1). A previous study reported that phenolic compounds, such as ellagic acid, phenolic acid, and flavonoids, are the major active compounds in *Duchesnea indica* [20–22]. Moreover, based on the results of the gas chromatography-mass spectrometry (GC-MS) analysis, the major components were as follows: gamma-sitosterol; hexadecanoic acid; linoelaidic check spacing acid; octadecanoic acid; 9, 12, 15-octadecatrienoic acid; and 2, 3-dihydro-3, 5-dihydroxy-6-methyl-(4H)-pyran-4-one (Table 2).

### 3.2. DIE Protects CFA-Induced Nitric Oxide Production and Cell Death in MH-S Macrophages

**Figure 2:**
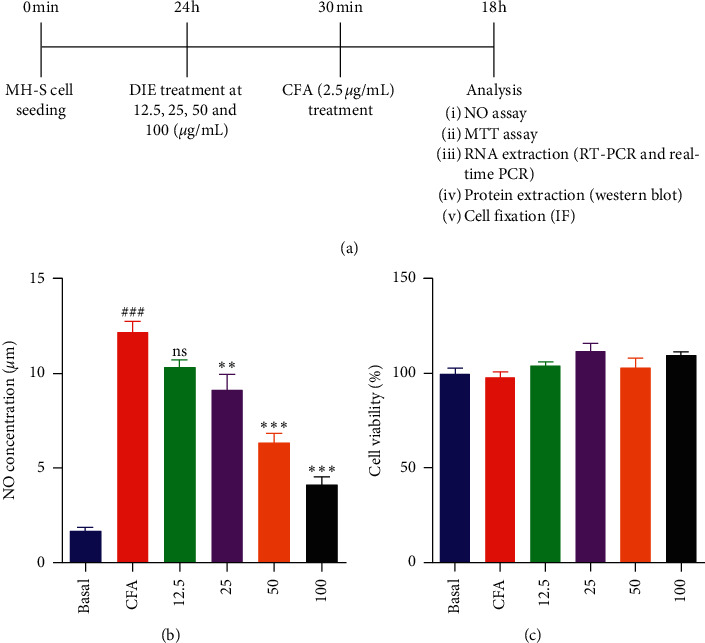
Effect of the *Duchesnea indica* extract (DIE) on coal fly ash- (CFA-) induced NO production and cell viability in murine alveolar macrophages (MH-S). (a) Schematic diagram of this study. The cells were in six groups including the basal (control) group, only CFA (2.5 *μ*g/mL) group, and CFA with the DIE (12.5, 25, 50, and 100 *μ*g/mL) group. Cells were treated with the above concentrations of the DIE for 30 min prior to the CFA treatment (2.5 *μ*g/mL) and incubated for 18 h. (b) NO level was determined using the Griess reagent method. (c) Cell viability assay was performed using the MTT reagent method. The cells were seeded in a 24-well plate. All values were expressed as standard error of mean ± (SEM) from three independent experiments. ^###^*p* < 0.001 when compared with basal group; ^*∗*^*p* < 0.05, ^*∗∗*^*p* < 0.01, and ^*∗∗∗*^*p* < 0.001 when compared with the only CFA-treated group.

Nitric oxide (NO) is an important mediator in the inflammatory condition, and excessive production of NO contributes to inflammatory diseases [23, 24]. In this study, the levels of NO in response to CFA in MH-S murine AM were measured using the Griess reaction method. The DIE potently inhibited the NO induction in a dose-dependent manner (Figure 2(a)). MTT assay was performed to determine the cell viability assay, and the results showed that the DIE did not affect the cell viability at the used concentration (Figure 2(b)). These results demonstrated that the DIE inhibited the NO production dose-dependently, and the used concentrations did not show cytotoxicity.

### 3.3. Preventive Effect of the DIE on CFA-Induced Proinflammatory Cytokines in MH-S Macrophages

**Figure 3:**
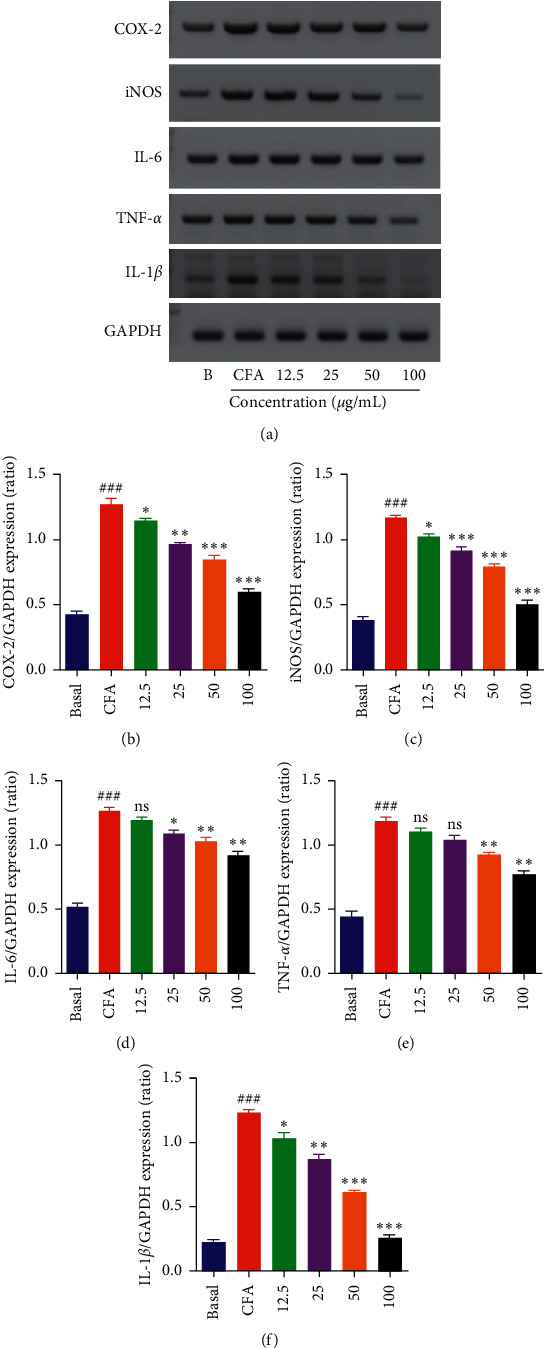
Effect of the DIE on CFA-induced proinflammatory mediators and cytokines in MH-S cells analyzed via RT-PCR. (a) After 18 h of incubation with CFA (2.5 *µ*g/mL), the mRNA levels of the proinflammatory mediators (COX-2 and iNOS) and proinflammatory cytokines (IL-6, TNF-*α*, and IL-1*β*) and GAPDH (housekeeping gene) were determined via reverse transcription-polymerase chain reaction (RT-PCR). (b–f) Densitometric analysis of relative mRNA expression levels which were quantified using the ImageJ program. The cells were seeded in a 6-well plate, and the doses of DIE were used as 12.5, 25, 50, and 100 *μ*g/mL. All values were expressed as standard error of mean ± (SEM) from three independent experiments. ^###^*p* < 0.001 when compared with the basal group; ^*∗*^*p* < 0.05, ^*∗∗*^*p* < 0.01, and ^*∗∗∗*^*p* < 0.001 when compared with the only CFA-treated group.

Pretreatment of MH-S with the DIE for 30 min decreased the levels of CFA-induced proinflammatory mediators and cytokines. The mRNA levels of proinflammatory mediators such as inducible nitric oxide synthase (iNOS) and cyclooxygenase-2 (COX-2) and proinflammatory cytokines including interleukin-6 (IL-6), IL-1*β*, and tumor necrosis factor-*α* (TNF-*α*) were analyzed using RT-PCR to investigate the anti-inflammatory properties of the DIE. The results showed that proinflammatory mediators and cytokines were dose-dependently reduced at the mRNA level (Figures 3(a)–3(f)). This finding suggested that the DIE downregulated the CFA-induced inflammatory cytokines and significantly reduced the mRNA levels mainly at concentrations of 50 and 100 *μ*g/ml.

### 3.4. DIE Ameliorates the CFA-Induced mRNA Expression of Proinflammatory Cytokines in MH-S Macrophages

**Figure 4:**
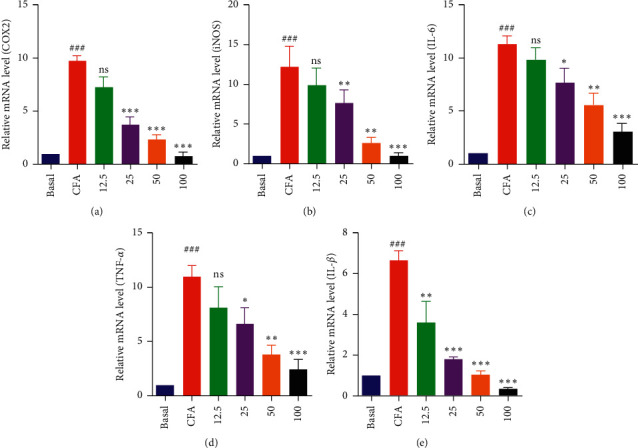
Effect of the DIE on CFA-induced mRNA expression in MH-S cells measured by real-time PCR. (a–e) After 18 h of incubation with CFA (2.5 *µ*g/mL), the mRNA levels of COX-2, iNOS, IL-6, TNF-*α*, and IL-1*β* were analyzed via quantitative real-time PCR. GAPDH was used as a control gene. The cells were seeded in a six-well plate, and the doses of DIE were used as 12.5, 25, 50, and 100 *μ*g/mL. All values were expressed as standard error of mean ± (SEM) from three independent experiments. ^###^*p* < 0.001 when compared with basal group; ^*∗*^*p* < 0.05, ^*∗∗*^*p* < 0.01, and ^*∗∗∗*^*p* < 0.001 when compared with the only CFA-treated group.

To confirm the results of the RT-PCR on the proinflammatory mediators and cytokines in MH-S cells, the mRNA expressions of the proinflammatory mediators were investigated and the proinflammatory cytokines were analyzed with real-time PCR. The mRNA expressions of the proinflammatory mediators and cytokines were noticeably dose-dependently downregulated with the DIE treatment (Figures 4(a)–4(e)). Taken together, the real-time PCR results suggested that the DIE reduced the inflammation in a concentration-dependent manner.

### 3.5. DIE Inhibits the Activation of NF-*κ*B Signaling in CFA-Treated MH-S Macrophages

**Figure 5:**
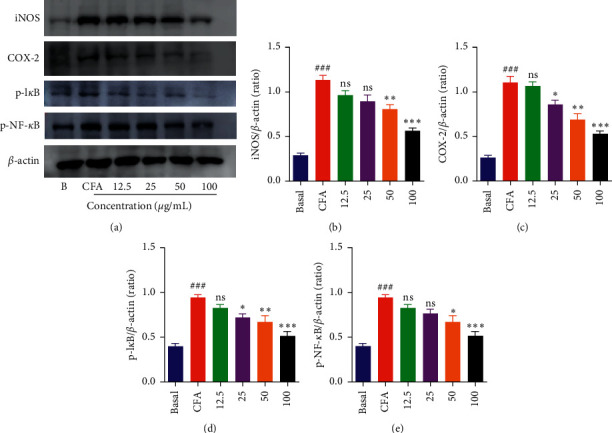
Effect of the DIE on CFA-induced NF-*κ*B phosphorylation in MH-S cells. (a) After 18 h of incubation with CFA (2.5 *µ*g/mL), the protein levels of iNOS, COX-2, p-I*κ*B, and p-NF*κ*B were determined using western blot. *β*-Actin was used as a loading control. (b–e) Densitometric analysis of protein expression levels which were measured using the ImageJ software. The cells were seeded in a six-well plate, and the doses of DIE were used as 12.5, 25, 50, and 100 *μ*g/mL. All values were expressed as standard error of mean ± (SEM) from three independent experiments. ^*∗*^*p* < 0.05, ^*∗∗*^*p* < 0.01, and ^*∗∗∗*^*p* < 0.001 when compared with the only CFA-treated group.

The effects of the DIE on nuclear factor-*κ*B (NF-*κ*B) signaling were measured as a transcription factor has an important role in inflammation. The activation with CFA leads to the inflammatory pathway, wherein NF-*κ*B signaling is a key axis in the inflammatory mechanism [1, 9]. Treatment with CFA significantly induced the phosphorylation of NF-*κ*B and inhibitor of kappa-B (I*κ*B), whereas the pretreatment of the DIE significantly inhibited the phosphorylation of NF-*κ*B and I*κ*B in MH-S cells (Figures 5(d) and 5(e)). This result suggested that the pretreatment with the DIE markedly downregulated the phosphorylation of the CFA-induced NF-*κ*B and I*κ*B activation in MH-S cells.

### 3.6. DIE Inhibits the Translocation of NF-*κ*B in CFA-Treated MH-S Macrophages

**Figure 6:**
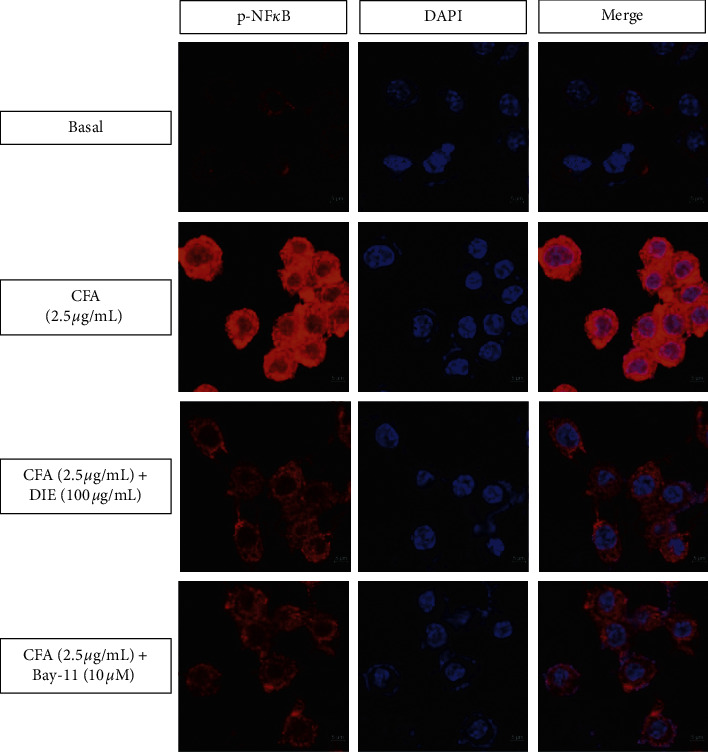
Effect of the DIE on CFA-induced NF-*κ*B translocation in MH-S cells. The cells were seeded on coated cover slip in a six-well plate, and the groups were as follows: basal group, only CFA (2.5 *μ*g/mL)-induced group, CFA with the DIE (100 *μ*g/ml) group, and CFA with Bay-11 (inhibitor of p-NF-*κ*B) group. The cells were treated with the DIE and Bay-11 (10 *µ*M) for 30 min prior to the CFA treatment (2.5 *μ*g/mL) and were incubated for 18 h. The nuclear translocation of p-NF-*κ*B was analyzed via immunofluorescence staining. The samples were mounted using ProLong™ Gold Antifade Reagent with DAPI to visualize the nuclei (blue). Stained cells were analyzed via confocal microscopy (ZEISS) at 1000x magnification.

**Figure 7:**
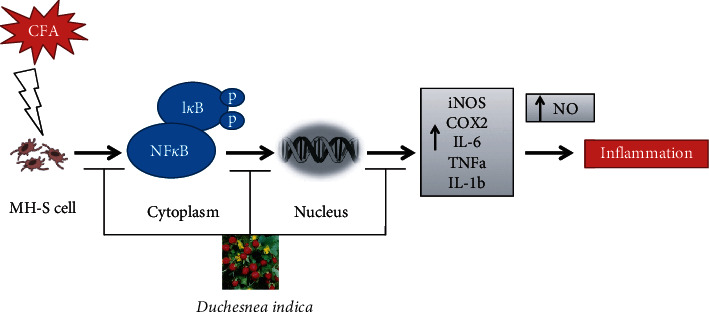
The mechanism of the DIE in CFA-induced inflammation in MH-S cells.

To find out whether the anti-inflammatory activities of the DIE are mediated by the NF-*κ*B signal transduction pathway in CFA-activated MH-S cells, the translocation of the activated p-NF-*κ*B from the cytoplasm to the nucleus was determined via immunofluorescence (IF) assay. The treatment with CFA increased the translocation of NF-*κ*B from the cytoplasm to the nucleus, whereas the treatment with highest dose of DIE (100 *μ*g/mL) significantly suppressed the nuclear translocation of p-NF-*κ*B in activated MH-S cells (Figure 6). Bay-11 was used as an NF-*κ*B inhibitor. The results of the IF assay indicated that the anti-inflammatory properties of the DIE were associated with its inhibitory effects on the phosphorylation of the NF-*κ*B signal pathway (Figure 7).

## 4. Discussion

*Duchesnea indica* has been widely used as a natural medicine in Asia especially in Bangladesh, China, Korea, and Japan for the prevention and treatment of numerous diseases, such as tissue inflammation, leprosy, congenital fever, and mainly cancer [12, 14]. Earlier studies have shown that its anti-inflammatory agents can suppress transcription factors, such as nuclear factor-*κ*B (NF-*κ*B) and function as a key regulator in inflammatory cascade [2, 7, 9]. In the present study, we examined the effects of *Duchesnea indica* on CFA-induced inflammation in the MH-S cell line.

Pharmacological studies have demonstrated that phenolic ingredients are the main bioactive compounds in the DIE [11]. Ellagic acid was indicated as the main compound of the DIE via the UPLC-QTOF-MS analysis. It is a phenolic compound and has been reported to exert a variety of biological properties, such as antioxidant, anti-inflammatory, and anticoagulant effects [25, 26]. Moreover, ellagic acid has also been shown to reverse hepatic damage by inhibiting the NF-*κ*B signaling pathway [27]. Previous findings are consistent with our study and suggest that ellagic acid could be one of the major active components responsible for any anti-inflammatory activity. The major compounds in the DIE were determined via GC-MS analysis: 2, 3-dihydro-3, 5-dihydroxy-6-methyl-(4H)-pyran-4-one; hexadecanoic acid; 9,12,15-octadecatrienoic acid; gamma-sitosterol; octadecanoic acid; and linoelaidic acid. Although the DIE has anti-inflammatory effects on CFA-induced inflammation in MH-S cells, further research is required to investigate the immune modulatory properties of the DIE in an animal model.

Particulate matter (PM) found in polluted air is becoming a major cause for health problems [28]. It has been reported that chronic exposure of PM is related to chronic inflammatory diseases especially severe lung diseases including chronic respiratory diseases, COPD, asthma, and mainly lung cancer [1, 29]. Inflammatory disorders cause excessive production of proinflammatory mediators and cytokines, which are the critical factors of many pathological and clinical manifestations [30]. Macrophages are the immune cells found in the immune regulatory system and responsible for upregulating the generation of inflammatory mediators.

Nitric oxide (NO) is generated by the iNOS in activated macrophages. NO is a small signaling molecule with essential roles in numerous body functions, but unregulated NO production can cause pathophysiological activities in the airway system [31, 32]. Such disturbances can lead to airway narrowing and lung hypersensitivity. The results showed that the DIE reduced NO production in CFA-induced MH-S cells, whereas NO production was markedly elevated in the only CFA-treated group. COX-2, which is another important proinflammatory mediator, plays a critical role in diverse inflammatory diseases [33]. In this study, it was found that the DIE suppressed the mRNA and protein expression levels of inflammatory mediators COX-2 and iNOS in CFA-activated MH-S cells.

Activated macrophages also generated other important proinflammatory cytokines, such as TNF-*α*, IL-6, and IL-1*β*, which are responsible for chronic inflammatory diseases and especially respiratory pathology [34]. IL-6 and TNF-*α* are the critical mediators of sepsis, and their uncontrolled regulation can cause cell death and DNA damage depending on the severity of cytokine production [9]. IL-1*β* increased the infiltration of inflammatory cells and mediated the activation of inflammatory cascade [35]. In the present study, the DIE inhibited the production of the IL-6, TNF-*α*, and IL-1*β* mRNA expressions in CFA-activated MH-S cells. In this study, the DIE also suppressed the protein expression of iNOS and COX-2, suggesting that the DIE has anti-inflammatory properties.

It is well established that the transcription factor, NF-*κ*B, is a key regulator for inflammation in stimulated macrophages. In normal conditions, NF-*κ*B is stable in the cytoplasm; however, upon CFA stimulation, activated cells result in the phosphorylation of I*κ*B. Hence, the NF-*κ*B is disrupted to induce the phosphorylation, and activated NF-*κ*B translocates into the nucleus from the cytoplasm [36, 37]. Thus, the p-NF-*κ*B then promotes the expression of proinflammatory mediators and cytokines. It has been previously shown that phosphorylated NF-*κ*B increases the transcription of inflammatory genes [38]. The western blot results demonstrated that the DIE dose-dependently reduced the CFA-stimulated I*κ*B and NF-*κ*B phosphorylation in the cytoplasm. Moreover, the results of the immunofluorescence staining indicated that CFA increased the p-NF-*κ*B translocation in the nucleus, where the DIE (100 *μ*g/mL) and Bay-11 (10 *μ*M) markedly inhibited the p-NF-*κ*B translocation from the cytoplasm to the nucleus. Based on our study, we speculated that the DIE might inhibit the phosphorylation of I*κ*B and NF-*κ*B resulting in the suppressive activity of the proinflammatory cytokines. In our study, only in vitro experiment was carried out, but in vivo excrement should be done to determine the exact mechanism of action of DIE as an anti-inflammatory agent.

## 5. Conclusion

In summary, the current study demonstrated the anti-inflammatory properties of the DIE in the CFA-induced MH-S cell line. The obtained data provide important information about CFA-activated inflammatory responses and suggest that the DIE could inhibit the expression of proinflammatory mediators and cytokines in MH-S cells. These findings demonstrate that the DIE could be used as a promising bioactive functional food for immune modulation, especially in the treatment of lung inflammatory diseases.

## Figures and Tables

**Table 1:** Primer used for RT-PCR and real-time PCR analysis.

RT-PCR	Forward primer sequences (5′–3′)	Reverse primer sequences (5′–3′)
COX-2	CACTACATCCTGACCCACTT	ATGCTCCTGCTTGAGTATGT
iNOS	CCCTTCCGAAGTTTCTGGCAGCAGC	GGCTGTCAGAGCCTCGTGGCTTTGG
IL-6	GTACTCCAGAAGACCAGAGG	TGCTGGTGACAACCACGGCC
TNF-*α*	TTGACCTCAGCGCTGAGTTG	CCTGTAGCCCACGTCGTAGC
IL-1*β*	CTGTGGAGAAGCTGTGGCAG	GGGATCCACACTCTCCAGCT
GAPDH	CACTCACGGCAAATTCAACGGCAC	GACTCCACGACATACTCAGCAC

*Real-time PCR*		
COX-2	GGCAGCCTGTGAGACCTTTG	GCATTGGAAGTGAAGCGTTTC
iNOS	GGCAGCCTGTGAGACCTTTG	GCATTGGAAGTGAAGCGTTTC
IL-6	TCCAGTTGCCTTCTTGGGAC	GTGTAATTAAGCCTCCGACTTG
TNF-*α*	TGCCTATGTCTCAGCCTCTTC	GAGGCCATTTGGGAACTTCT
IL-1*β*	CAACCAACAAGTGATATTCTCCATG	GATCCACACTCTCCAGCTGCA
GAPDH	CACTCACGGCAAATTCAACGGCAC	GACTCCACGACATACTCAGCAC

**Table 2:** Major compounds in the *Duchesnea indica* extract identified via GC-MS analysis.

Compounds	Area (%)
2,3-Dihydro-3,5-dihydroxy-6-methyl-(4H)-pyran-4-one	20.37
Hexadecanoic acid	18.04
9,12,15-Octadecatrienoic acid	11.1
Gamma-sitosterol	7.25
Octadecanoic acid	5.52
Linoelaidic acid	4.54

## Data Availability

All data generated during this study are included within this manuscript.
